# Enhancement of Single Molecule Raman Scattering using Sprouted Potato Shaped Bimetallic Nanoparticles

**DOI:** 10.1038/s41598-019-47179-4

**Published:** 2019-07-24

**Authors:** R. V. William, G. M. Das, V. R. Dantham, R. Laha

**Affiliations:** Department of Physics, Indian Institute of Technology Patna, Bihta, 801103 India

**Keywords:** Nanoparticles, Nanophotonics and plasmonics

## Abstract

Herein, for the first time, we report the single molecule surface enhanced resonance Raman scattering (SERRS) and surface enhanced Raman scattering (SERS) spectra with high signal to noise ratio (S/N) using plasmon-active substrates fabricated by sprouted potato shaped Au-Ag bimetallic nanoparticles, prepared using a new one-step synthesis method. This particular shape of the nanoparticles has been obtained by fixing the amount of Au and carefully adjusting the amount of Ag. These nanoparticles have been characterized using scanning electron microscopy, extinction spectroscopy, and glancing angle X-ray diffraction. The single molecule sensitivity of SERS substrates has been tested with two different molecular Raman probes. The origin of the electromagnetic enhancement of single molecule Raman scattering in the presence of sprouted shape nanoparticles has been explained using quasi-static theory as well as finite element method (FEM) simulations. Moreover, the role of (i) methods for binding Raman probe molecules to the substrate, (ii) concentration of molecules, and (iii) Au-Ag ratio on the spectra of molecules has been studied in detail.

## Introduction

Single molecule Surface enhanced Raman spectroscopy has become an interesting tool for quick identification of toxic substance, understanding molecular dynamics, the real-time detection and extraction of chemical structure of single molecules at their native state^1–3^. It is important to note that obtaining single molecule Raman scattering spectra is extremely difficult task due to the infinitesimal scattering cross section^4^. So far, only a few researchers have succeeded in obtaining single molecule Raman spectra by placing the molecules at the hotspots of plasmonic particles in surface enhanced Raman scattering (SERS) substrates^5,6^. However, all such reported spectra visibly suffer from very low signal to noise ratio (S/N). Therefore, there is tremendous demand to fabricate efficient SERS substrates for enhancing the S/N of Raman spectra of single molecules.

Recently, bimetallic nanoparticles have been shown to be technologically superior to the corresponding monometallic counterpart due to their controlled composition, size and shape^7^. Commonly, bimetallic nanostructures are synthesized using Au and Ag. Due to similar lattice parameters (Au-4.08 Å; Ag-4.09 Å) with face-centered cubic crystal structure, preparation of Au-Ag bimetallic nanostructures is more convenient as compared to other bimetallic nanostructures such as Cu-Ag, Au-Pd, and Ag-Pt, etc^8–11^. The synergic effect of bimetallic nanostructure is very much expected to attain strong electromagnetic field enhancement as compared to the individual plasmonic responses.

Fan *et al*. have compared the SERS performance of both monometallic and bimetallic (Au-Ag) nanostructures with four different Raman probes, and found that the SERS detection limit of bimetallic nanoparticles is 10 µM. Also, from density functional theory, it was concluded that the SERS signal strength depends on the binding site in the SERS substrate as well as nature of Raman probe molecules^12^. Akshaya *et al*. have synthesized Au-Ag bimetallic core–shell–satellite nanoparticles and demonstrated the detection limit of 10^−9^ M in case of 1-Naphthalenethiol^13^. Likewise, researchers have developed many strategies for preparing bimetallic core-shell nanostructures and achieved the highest SERS detection limit of 10^−9^ M using thiram as Raman probe^14^. Au-Ag core-shell nanoparticles have also been widely investigated in recent years for studying several bioanalytes as Raman probes. Apart from bimetallic core-shell nanoparticles, Au-Ag bimetallic flowers (Ag-stem and Au-core) have also been fabricated and the corresponding SERS detection limit has been reported to be of 10^−6^ M^15,16^.

**Figure 1:**
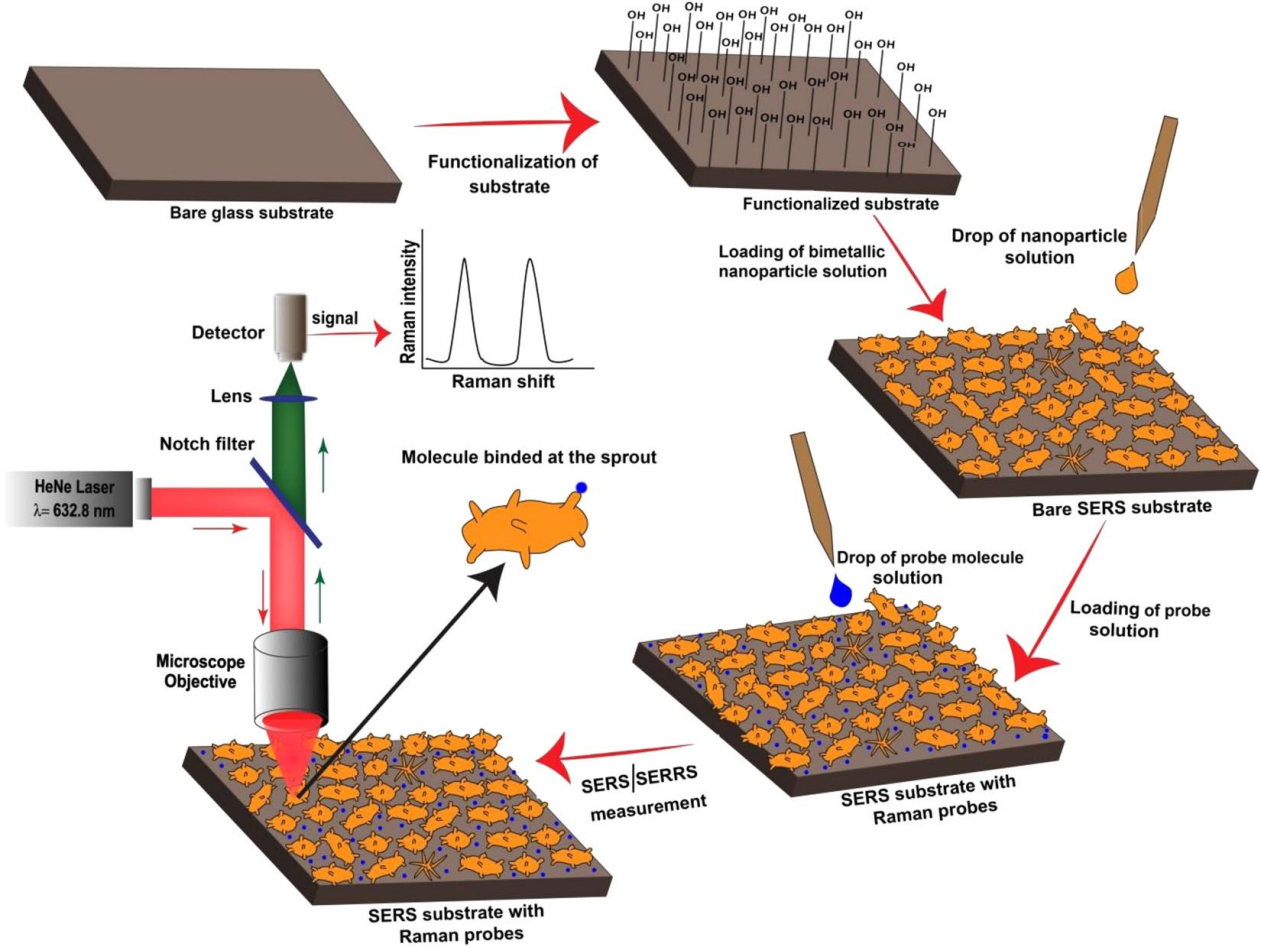
The flow diagram for SERRS and SERS spectral measurements of different Raman probe molecules using synthesized Au-Ag nanopariticles.

It is important to note that even though bimetallic nanostructures have been reported to be better than the corresponding monometallic ones; the SERS detection limit achieved so far is 10^−9^ M^17,18^. In present work, we have successfully brought the detection limit down to 10^−15^ M. Herein, we report (i) the synthesis and characterization of complex shaped Au-Ag bimetallic nanoparticles, (ii) SERRS and SERS spectra of single molecules of methylene blue (MB) and crystal violet (CV) respectively using the synthesized bimetallic nanoparticles, and (iii) the role of methods for binding Raman probe molecules to the substrate, concentration of molecules, and Au-Ag ratio on spectra of molecules and (iv) numerical simulations based on finite element method (FEM) to support the experimental findings. The complete scheme of the procedure starting from glass plate cleaning to collecting the SERS signal at detector is shown schematically in Fig. 1.

## Results and Discussion

### Morphology study using field emission scanning electron microscope

**Figure 2:**
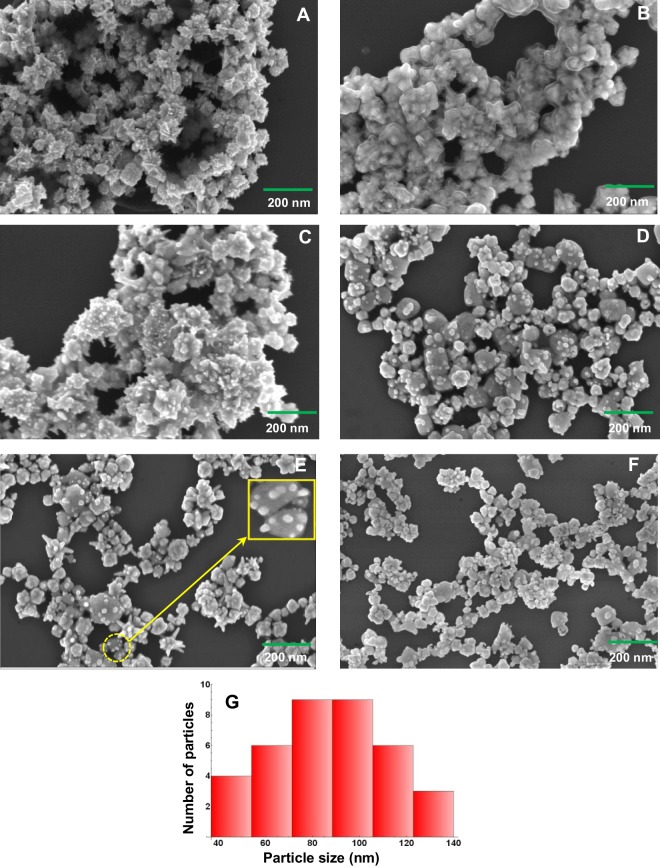
FESEM images of bimetallic nanoparticles prepared with different Au-Ag ratios (**A**) 10:1, (**B**) 10:2, (**C**) 10:3, (**D**) 10:6, (**E**) 10:7, and (**F**) 10:10. Inset in panel E shows magnified image of a few nanoparticles shown in circle. Panel G represents histogram showing particle size distribution obtained from panel E.

Field Emission Scanning Electron Microscope (FESEM) images were recorded for all the synthesized bimetallic nanoparticles by dispering the nanoparticles over silicon substrates. Figure 2A–F shows the FESEM images of the bimetallic nanoparticles with Au-Ag ratio 10:1, 10:2, 10:3, 10:6, 10:7 and 10:10. For all the Au-Ag ratio from 10:1 to 10:5 (10:5 images were not mentioned in Fig. 2), the nanoparticles are formed with spikes representing star shaped structure. With the increase in ratio from 10:5 to 10:6, the sharpness of the spikes gets reduced and the nanoparticles resemble to sprouted potato shaped structure, as shown in Fig. 2D. Moreover, the shape of the sprouts and number of sprouts were found to increase when the ratio was increased further. Thus, the formation of Au-Ag bimetallic nanopartcles was found to be fully dependent on the proportion of silver ions added in each steps. Here, it is also worth to mention that in the previous work, the star shaped nanoparticles were observed for Au-Ag ratio ranging from 6:2 to 100:2 and pop corn shaped nanoparticles were obatined when the ratio was increased to 200:2^19^. However, in our present work, the complex shaped nanostructures like stars (10:1 to 10:5) and sprouted potato (10:6 to 10:10) were formed. As it will be shown later that among all the ratios, 10:7 shows best Raman enhancement, the dimensions of the nanoparticles involved in 10:7 ratio are crucial for analysis. Therefore, the mean size of nanoparticles has been estimated for 10:7 and the size distribution has been shown as histogram in Panel G of Fig. 2. The mean size has been found to be around 90 nm.

### UV-Visible spectra

**Figure 3:**
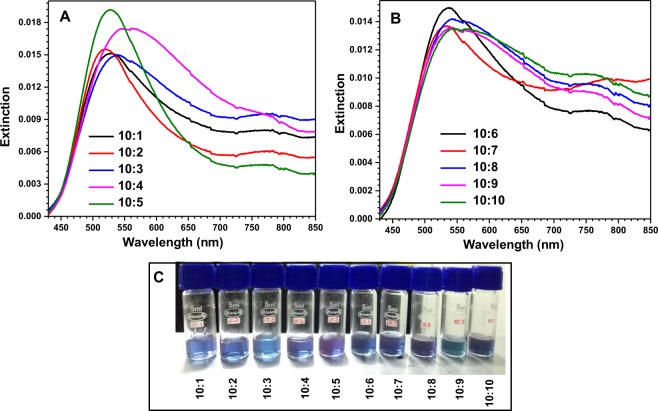
Panel (A,B) combinedly represent the extinction spectra of Au-Ag bimetallic nanoparticles with different Au-Ag ratios. Panel (C) shows images of syntheized Au-Ag bimetallic nanoparticle solutions with different proportions.

Figure 3 shows the optical results and optical images of solutions. According to electromagnetic theory of SERS, the strength of SERS signal directly depends upon the local field of nanoparticles. This local field can be maximized easily when the incident laser wavelength matches with the localized surface plasmon resonance (LSPR) wavelength of the nanoparticles^20–22^. For this purpose, we need to have the knowledge regarding LSPR wavelength of synthesized nanoparticles. In general, the LSPR wavelength can be predicted from the optical absorption/extinction spectrum of nanoparticles^23,24^. Therefore, the extinction spectra of all synthesized Au-Ag bimetallic nanoparticles were recorded and are shown in Fig. 3A,B. The peak in every spectrum represents LSPR and is found to be sensitive with each ratio due to the variation in the morphology. Figure 3C shows images of prepared solutions of Au-Ag nanoparticles with different Au:Ag ratios. Initially, for 10:1 ratio, it appears blue in colour and with the increase in ratio, the colour of the nanoparticle solution changes significantly.

From Fig. 3A,B, it is clear that the entire LSPR band falls in the visible region so that these nanoparticles will be useful for SERS. Moreover, broad spectra are found in the case of sprouted potato (SP) shaped nanoparticles as compared with star shaped nanoparticles. The broad spectra are very useful in the case of SERS experiments because, these nanoparticles are able to enhance the electric fields of laser light as well Raman scattering light of different frequencies. In other words, these nanoparticles will be useful for obtaining all possible Raman modes of various samples in Raman spectra. Also, the broadness of individual spectrum was found to increase with the Au-Ag ratio. The difference in intensity of the peaks in these spectra might be due to the variation of extinction cross section which depends upon the polarizability of the nanoparticles, and concentration of the nanoparticle solutions^25,26^.

### XRD analysis

**Figure 4:**
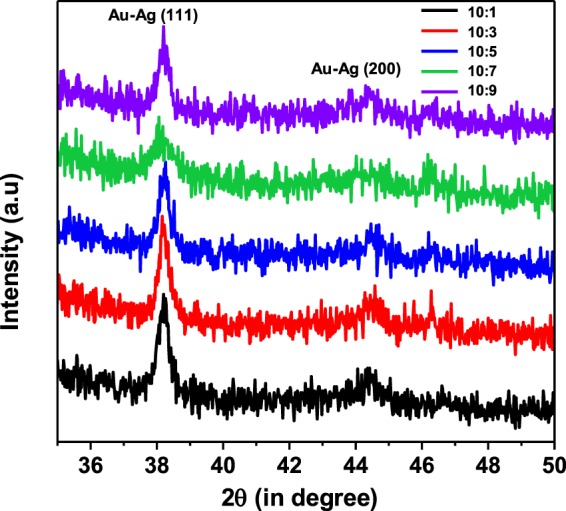
GXRD patterns of bimetallic nanoparticles with different Au-Ag ratios.

Figure 4 shows the glancing angle X-ray diffraction (GXRD) patterns of bimetallic nanoparticles with different Au-Ag ratios. In order to get the XRD peaks with non-negligible intenstity in GXRD measurements, the amount of the nanoparticle solution dropcasted on to glass plate was increased from 10 µl to 20 µl. It is apparent from Fig. 4 that two peaks at 38.2° and 44.5° are prominent for all the ratios. As per JCPDS (file nos 01-071-3762 and 01-089-3697), the obatined peaks correspond to (111) and (200) planes of Au/Ag. It is important to note that the positions of peaks due to Au and Ag differ very little (~0.08° for 111 orientations) because of their lattice constants being very close (4.08 Å and 4.09 Å for Au and Ag respectively). For this reason, XRD patterns for Au-Ag bimetallic nanostructures are commonly represented by those of Au or Ag^27,28^.

### SERS activity of different bimetallic nanoparticles

**Figure 5:**
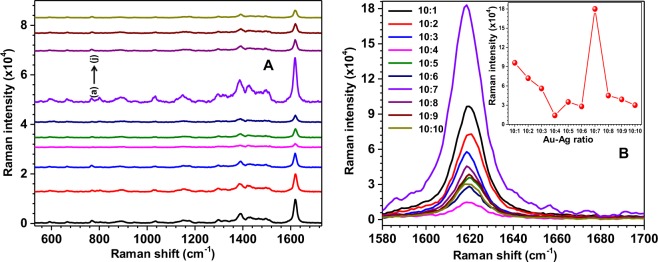
Panel (A) shows the SERRS spectra of MB obtained with the bimetallic nanoparticles of different Au-Ag ratios (a) 10:1, (b) 10:2, (c) 10:3, (d) 10:4, (e) 10:5, (f) 10:6, (g) 10:7, (h) 10:8, (i) 10:9, and (j) 10:10. All spectra are offset for better clarity. Panel (B) corresponds to the Raman peak at 1622.1 cm^−1^ alone (without offset). Inset of panel B represents the variation of intensity of the Raman peak at 1622.1 cm^−1^ for different Au-Ag ratios.

To study the SERS activity of Au-Ag bimetallic nanostars and the role of Au-Ag ratio on SERS signal, the MB molecules were chosen. The chemical formula of MB is C_16_H_18_ClN_3_S with molecular weight of 319.851 g/mol. It is a biocompatible material and widely used in antimicrobial photodynamic therapy^29^. To investigate the role of Au-Ag ratio on SERS signal, the SERS substrates on which the Raman probe molecules were dispersed by drop casting of 10 µl of MB solution of 10^−7^ M, were preferred. Since MB shows strong absorption from 610–660 nm due to electronic transitions, the Raman spectra of MB adsorbed on Au-Ag bimetallic substrate excited at 632.8 nm laser can be called as surface enhanced resonance Raman spectra (SERRS)^30,31^. Figure 5A shows the SERRS spectra of MB molecules obtained with the bimetallic nanoparticles of different Au-Ag ratios. In this figure, it is apparent that all possible Raman peaks of MB molecules are observed and their Raman frequencies were found same for all Au-Ag ratios.

The observed Raman peaks at 591.9, 662.9, 769.5, 889.5, 1033.7, 1148.3, 1298.1, 1386.4, 1428.6, 1498.3 and 1622.1 cm^−1^ of MB molecules agree well with the standard literature^32,33^ and the details of the assignment of modes are described in Table 1. The image of molecular structure for MB (created using MOLVIEW software) is shown in Supplementary Fig. S1. The Raman peak at 1622.1 cm^−1^ (C-C ring stretching of MB) is relatively dominant in all ratios. Panel B of Fig. 5 shows the Raman peak at 1622.1 cm^−1^ alone (without offset) and its inset shows the variation in the intensity of Raman peak corresponds to C-C ring stretching with different Au-Ag ratios. It is clear that the strength of SERRS signal of MB molecules is more in the presence of SERS substrates fabricated with the bimetallic nanoparticles with Au-Ag ratio 10:7 due to several factors like composition, distribution of sprouts and possibility for a molecule being found at a “hot spot”. Therefore, this study motivated us to use the SERS substrates fabricated with bimetallic nanoparticles with Au-Ag ratio 10:7 for capturing single molecule SERRS spectra.

**Table 1:** Assignment of Raman modes to the observed Raman shifts for MB.

Observed Raman shift (cm^−1^)	Mode assignments
591.9	Skeletal deformation of C-S-C
662.9	In-pane bending of C-C ring
769.5	In-pane bending of C-H
800.1	Stretching of C-H
889.5	In-plane bending of C-H
1033.7	In-plane bending of C-H and C-S
1148.3	In-plane bending of C-H
1298.1	In-plane ring deformation of C-H
1386.4	Symmetrical stretching of C-N
1428.6	Asymmetrical stretching of C-N
1498.3	Asymmetrical stretching of C-C
1622.1	Ring stretching of C-C

### Role of methods for binding and number density of Raman probe molecules on SERRS signal

**Figure 6:**
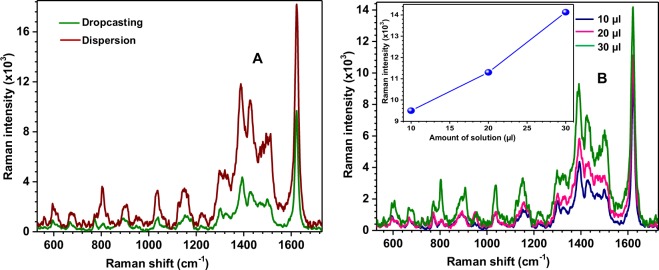
Panel (A) represents SERRS spectra of MB molecules (nM) attached to SERS substrates using different methods. Panel B represents variation in SERRS spectra with amount of MB solution (number density of MB molecules) of nM. The inset plot in panel (B) represents the variation of Raman intensity with the amount of MB solution.

Two different methods were used for binding the Raman probe molecules to the SERS substrates. In the first method, the MB molecules were dispersed over the SERS substrates by drop-casting of the MB solution of nanomolar (nM) concentration, as described in the experimental section. In the second method, the MB solution was added to the synthesized parent bimetallic nanoparticle solutions and kept aside for 10 minutes allowing the MB molecules to bind the nanoparticles. After this, 10 µl of the new mixture of solutions was drop-casted on to glass substrate and dried inside a desiccator for 30 minutes. The SERRS spectra were recorded separately for both types of SERS substrates and obtained spectra are shown in Panel A of Fig. 6. From this figure, it is apparent that the SERRS signal intensity is relatively better for the molecules attached to the SERS substrates using the second method as described above.

The increment in the SERRS signal in this case represents that relatively more number of molecules are adsorbed on the surface of the nanoparticles^34,35^. Thus, it can be easily concluded that the SERRS signal strength also depends upon the method adopted for attaching molecules to the SERS substrates. To understand the dependence of SERRS signal strength on the number density of MB molecules experimentally, the SERRS spectra of molecules were recorded by dispersing them over SERS substrates by drop-casting of 10 µl, 20 µl and 30 µl of MB solutions of nM and the obtained spectra are shown in Panel B of Fig. 6. Inset of Panel D shows the linear dependence of Raman intensity with the number density of molecules over the SERS substrate which is consistent with the SERS theory.

### Single molecule spectra of MB and CV molecules

**Figure 7:**
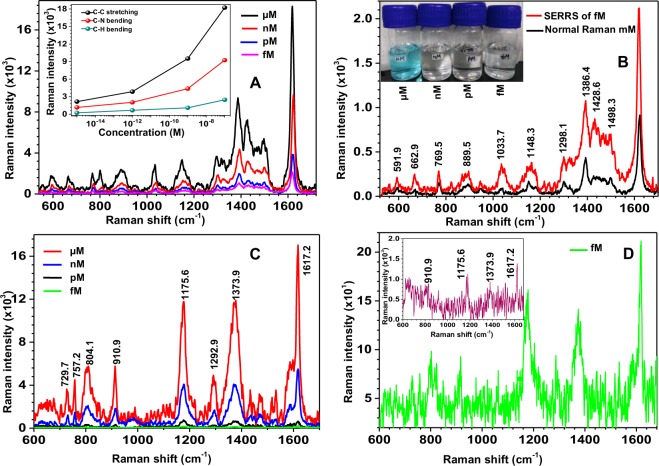
Panel (A) represent SERRS spectra of MB molecules dispersed over SERS substrate by drop-casting of 10 µl of MB solutions of different concentrations. Inset of panel (A) shows intensity variation of prominent Raman modes of MB. Panel (B) shows SERRS spectra of MB molecules for fM concentration and normal Raman spectrum (without SERS substrate) of MB in mM concentration. Inset of Panel B represents image of MB solutions at different concentrations. Panel (C) represents SERS spectra of CV molecules drop-casted using 10 µl of CV solutions of different concentrations. Panel (D) shows SERS spectra of CV molecules for fM concentration and inset of panel (D) represents normal Raman spectra of CV molecules at mM.

Panel A of Fig. 7 represents the SERRS spectra of MB molecules dispersed over SERS substrates by drop-casting of 10 µl of MB dye solutions of concentrations 10^−7^, 10^−9^, 10^−12^ and 10^−15^ M. The reduction of concentration of the MB solution was done gradually by a step of one order of concentration, after observing all the allowed Raman modes for MB with high S/N at each step. The actual images of the solutions with different lower concentrations are shown as the inset of panel B of Fig. 7. The SERRS spectrum obtained with the femtomolar (fM) solution alone is separately shown in panel B of Fig. 7, in order to realize the high S/N even for fM concentration. Also, for the sake of comparison, a normal Raman spectrum of MB in millimolar (mM) concentration is given in the panel B of Fig. 7. It can be seen that all the expected Raman modes were visible in the normal Raman spectrum, with most of the peaks being visibly weak when compared to the SERRS spectrum. It is important to note that in the existing reports, the typical concentration of the sample (or dye solution) used for single molecule SERS measurements is in the order of picomolar (pM)^36–41^. However, in the present case, the lowest concentration of the sample taken for SERRS is fM. Conventionally, a femtomolar solution represents the case of single molecules^42–44^. Thus, we can safely conclude that the obtained SERRS spectra for the case of femtomolar concentration correspond to single MB molecules. Based on the high S/N of the SERRS spectrum shown in panel B of Fig. 7, it is easy to predict that the lowest limit of SERS detection is much lower than the fM. Hence, the fabricated substrates are efficient for studying single molecule SERRS. The inset of panel A of Fig. 7 shows the variation in intensity of specific Raman modes at 1622.1, 1386.4 and 889.5 cm^−1^ with sample concentration.

The single molecule sensitivity of SERS substrate has also been tested with crystal violet (CV) molecules. Panel C of Fig. 7 represents the SERS spectra of CV molecules dispersed over SERS substrates by drop-casting of 10 µl of CV dye solutions of concentrations 10^−7^, 10^−9^, 10^−12^ and 10^−15^ M. The observed Raman peaks at 1617.2, 1373.9, 1292.9, 1175.6, 910.9, 804.1, 757.2, 729.7 cm^−1^ of CV molecules agree well with the standard literature and the details of the assignment of modes are described in Table 2^45–47^. Also, the image of molecular structure for CV is shown in Supplementary Fig. S2. The SERS spectrum obtained with the fM solution alone is separately shown in panel D of Fig. 7 for better clarity, along with the normal Raman spectrum for CV at mM concentration given in the inset. It is clear that the signal in normal Raman spectrum of CV shows poor S/N, with many peaks being not even visible when compared to the SERS spectrum. From panel D of Fig. 7, it is apparent that the fabricated SERS substrates are sensitive enough for single molecule detection. These substrates can also work for studying SERS spectra of single protein molecules which are useful for the Research community for the detection of dangerous diseases at very early stage, as well as for predicting the structural information of new molecules which is useful for developing drugs^48–50^.

**Table 2:** Assignment of Raman modes to the observed Raman shifts for CV.

Observed Raman shift (cm^−1^)	Mode assignments
729.7	C-N-C symmetric stretching vibration of Dimethyl amino group
757.2	C-H ring vibration
804.1	C-H ring vibration
910.9	C-H out of plane bending mode
1175.6	C-H in plane bending mode
1292.9	C-H ring vibration
1373.9	Stretching vibration of Nitrogen and Phenyl ring
1617.2	In-plane aromatic C-C stretching vibration

### Theoretical & numerical aspects of SP shaped nanoparticles

As mentioned in introduction, obtaining single molecule Raman spectra with high S/N is entirely difficult task due to infinitesimal scattering cross section. However, in the present case, we have successfully obtained single molecule (10^−15^ M) spectra with high S/N ratio. The strong single molecule SERRS/SERS signal in the present case is mainly due to electromagnetic enhancement and partially due to the chemical enhancement. The reason for the giant electromagnetic enhancement of single molecules adsorbed on the sprouted potato shaped nanoparticles can be understood using quasi-static theory as follows.

**Figure 8:**
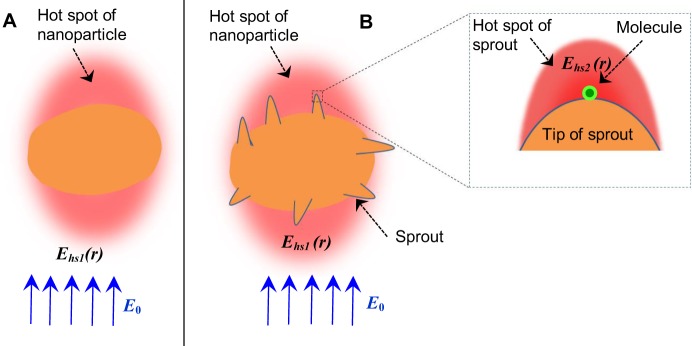
Panel (A,B) illustrates hotspots on the surface of sprouts-free and sprouted potato shaped nanoparticles, respectively. The zoomed portion in Panel (ii) shows single molecule adsorbed at the tip of a sproute. ***E***_0_ is the electric fied of incident light. ***E***_*hs1*_(***r***) is the electric field at the hot spot of sproutes-free potato shaped nanoparticle and this electric field remains same even in the presence of sproutes (except at the tips of sproutes). ***E***_*hs2*_(***r***) is the electric field at the hot spot of one sprout.

Panel A of Fig. 8 shows the schematic of hot spots generated on the surface of fresh potato shaped nanostructure in the presence of external electric field. According to the quasi-static theory, the electric field at any of the hot spots of potato shaped nanostructure is given by^51^.

1 $${{\boldsymbol{E}}}_{{\bf{hs1}}}={{\boldsymbol{E}}}_{{\bf{0}}}+{\eta }{\chi }{{\boldsymbol{E}}}_{{\bf{0}}}$$

where ***E***_0_ is the applied electric field, *η* is resonant enhancement factor and χ is known as the non-resonant enhancement factor. The factor *η* is due to the resonant excitation of the localized surface plasmons inside nanostructure. Whereas, *η* depends upon the size, shape, and relative electric permittivity of the nanostructure, χ depends only upon the geometry of the nanostructure. For the case of elongated nanostructures or for nanostructures having curved surfaces, χ dominates over *η* due to the lightning rod effect. The enhancement of Raman scattering signal of single molecule (ξ_Raman_) adsorbed at any of the hot spot of potato shaped nanostructure is given by^52^,

2 $${\xi }_{Raman1}={|\frac{{{\boldsymbol{E}}}_{{\boldsymbol{hs1}}}}{{{\boldsymbol{E}}}_{{\boldsymbol{0}}}}|}^{4}={(1+\chi \eta )}^{4}$$

Panel B of Fig. 8 shows the schematic of hotspots on the surface of sprouted potato shaped nanostructure. The electric field of hot spot on the surface of any of the sprouts is given by

3 $${{\boldsymbol{E}}}_{{\boldsymbol{hs2}}}={{\boldsymbol{E}}}_{{\boldsymbol{hs1}}}+\eta ^{\prime} {\chi }^{\prime} {{\boldsymbol{E}}}_{{\boldsymbol{hs1}}}$$

where, *η*′ and *χ*′ have same meaning and physical significance as those of the respective unprimed counterparts, except the fact that primed symbols represent the corresponding factors for the sprout. Similarly, whereas *η*′ depends upon the size, shape, and relative electric permittivity of the sprout, *χ*′ depends only upon the geometry of the sprout. After incorporating Eq. (1), Eq. (3) modifies as

4 $${{\boldsymbol{E}}}_{{\boldsymbol{hs2}}}=(1+\chi \eta +\chi ^{\prime} \eta ^{\prime} +\chi \eta \chi ^{\prime} \eta ^{\prime} ){{\boldsymbol{E}}}_{{\boldsymbol{0}}}$$

The enhancement of Raman scattering signal of molecule (ξ_Raman2_) adsorbed at any of the hot spot of sprout is given by^53^

5 $${\xi }_{Raman2}={|\frac{{{\boldsymbol{E}}}_{{\boldsymbol{hs2}}}}{{{\boldsymbol{E}}}_{{\boldsymbol{0}}}}|}^{4}={(1+\chi \eta +\chi ^{\prime} \eta ^{\prime} +\chi \eta \chi ^{\prime} \eta ^{\prime} )}^{4}$$

Since, the values of *χ*, *η*, *χ*′ and *η*′ are larger than 1.0, the value of ${\xi }_{Raman2}$ is significantly larger than ${\xi }_{Raman1}$. This means that the nanoparticles with sprouts are extremely useful for enhancing the SERS of single molecule. It is worth to mention here that the Eq. (5) assumes no plasmon coupling^54,55^ between the sprouts. It is well known that size, shape and aggregation of particles lead to electric field enhancements differently^56^. In addition, if there is plasmon coupling between sprouts, then the electric field gets enhanced significantly further at the nanogaps between the sprouts due to the constructive overlapping of plasmon modes. The importance of shape in the present work can be realized when the morphology of the prepared nanoparticles and the corresponding SERS signal is compared. From the SEM images, it can be seen that the ratios 10:6 and 10:7 show similar features as far as the shape (sprouts) is concerned. But the observed SERRS signal due to 10:7 is about 7 times higher than that observed for 10:6 (Fig. 5B). This can be explained in the following way.

**Figure 9:**
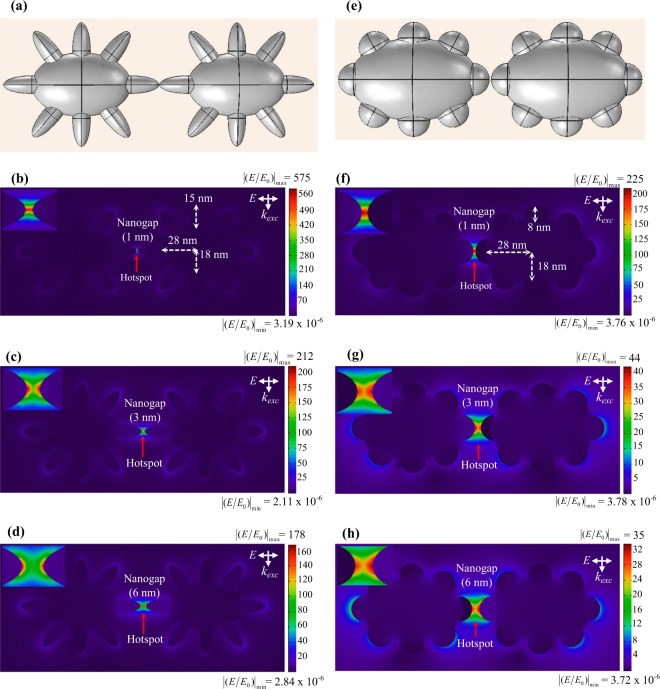
Panel (a) shows the modelling of potato shaped nanoparticles with sharp sprouts, separated by a few nanometers. Panels (b–d) represent the local field distribution of nanoparticles (shown in Panel (a)) with different separations. Panel (e) shows the modelling of potato shaped nanoparticles with blunt sprouts, separated by a few nanometers. Panels (f–h) represent the local field distribution of nanoparticles (shown in Panel (e)) with different separations. Here, all the numerical simulations have been done in air medium, λ = 633 nm, E_0_ = 1 V/m. Insets show the electric field enhancement at the nanogaps.

The sprouts in case of 10:7 are sharper as compared to 10:6. A close look at all the SEM images along with the SERS enhancements indicates that enhancement is very high for the particles with sharp sprouts, combined with a clustering that favors proper hotspot formation. This explanation was further vindicated by numerical simulations given in Fig. 9. The result of simulations clearly demonstrates the effect of shape and interparticle coupling on the hotspot intensity. Taking clue from the variation in shapes observed in SEM images, the simulations were performed particularly for potato shaped nanoparticles with blunt and sharp sprouts. When Fig. 9(c,f) are compared, it can be seen that sharp sprouts give rise to electric field enhancement (E/E_0_) which is 5 times higher than the enhancement by blunt sprouts. Similarly, comparison of Fig. 9(a,c) indicates that spikes oriented and close to each other give rise to higher enhancements values. It is important to mention here that the enhancement values observed in numerical simulations are maximum values for the simulated nanostructures which are the average representations from the experimental SERS substrates. These values need not be translated directly into experimental enhancement values, because of the obvious fact that the electric field strength at all nanogaps available in SERS substrate may not be uniform due to the variation of nanogap size, shape and size of the nanostructures. In addition, location and number density of the Raman probe molecules, and orientation of nanostructures with respect to the incident electric field also play important role in the SERS enhancement.

## Conclusion

We have successfully fabricated the SERS substrates containing Au-Ag bimetallic nanoparticles, with Au-Ag ratio varied from 10:1 to 10:10 using a new wet chemical method. The star shaped nanoparticles have been observed for Au-Ag ratios from 10:1 to 10:5. However, the sprouted potato shaped nanoparticles have been found for the ratio 10:6. Also the number and sharpness of sprouts were found to increase with the further increase in Ag content. The SERS substrates prepared with bimetallic nanoparticles of Au-Ag ratio 10:7 have been found to be relatively better in enhancing the SERRS signal. Using these substrates, the SERRS spectra of single MB and SERS spectra of single CV molecules have been recorded with high S/N. From the obtained single molecule SERRS spectra, the limit of detection has been found to be lower than fM. The origin of the enhancement has been explained with the help of quasi-static theory and supported by numerical simulations. It has been found that the strength of single molecule SERS signal also depends on the way the Raman probe molecules are attached to the SERS substrate. Mixing the solutions of nanoparticles and Raman probe molecules prior to drop-casting was found to help in achieving higher S/N. Similarly, SERRS/SERS intensity increases significantly with the number density of Raman probe molecules. To the best of our belief, this report will help the SERS community for fabricating efficient SERS substrates using simple and cost-effective approach for studying SERS of single dye molecules as well as protein molecules for (i) obtaining structural information and (ii) early stage disease detection.

## Experimental Details

### Chemicals required

The chemicals used were Hydrogen tetrachloroaurate (III) trihydrate (HAuCl_4_.3H_2_O, ≥99.9%)), silver nitrate (AgNO_3_, ≥99.9%), ascorbic acid C_6_H_8_O_6_ (AA, ≥99.9%), hydrogen peroxide (H_2_O_2_, ≥99.9%), sulphuric acid (H_2_SO_4_, ≥99.9%) and MB (≥99.9%). All the chemicals were procured from Sigma-Aldrich and were used with as received purity. During the whole synthesis process, ultrapure water was used as obtained from a Millipore water purification system (Milli-Q).

### Synthesis of Au-Ag bimetallic nanoparticles

The bimetallic nanoparticles were prepared using suitable modification of sequences of the synthesis route prescribed by Cheng *et al*.^19^. In order to prepare bimetallic nanoparticles with different Au-Ag ratios, HAuCl_4_ and AgNO_3_ solutions were prepared seperately at 10 mM concentration each. At first, HAuCl_4_ and solution were added together in 1 ml of Milli-Q water at 10:1 ratio followed by the addition of 4 µl of AA of 100 mM. AA was added quickly in order to avoid the precipitation of Ag as AgCl. A change of colour in the form of intense blue from colourless was observed in few seconds, indicating the formation of Au-Ag bimetallic nanoparticles. Here, AA acts as a reducing agent for Au and Ag. Then, the ratio of Au-Ag was varied from 10:1 to 10:10 by fixing the amount of Au and varying amount of Ag. The transformation of color of nanoparticle solution has been observed with Au-Ag ratio. Cheng *et al*. have prepared Au-Ag star-shaped bimetallic nanoparticles by keeping the overall concentration of AgNO_3_ constant and varying the proportion of HAuCl_4_ and AA. However, in the present work, we have varied only the amount of AgNO_3_, keeping the quantities of other two chemicals fixed. The amount of AgNO_3_ was varied from 2 µl to 20 µl, with the amounts of HAuCl_4_ and AA being fixed at 20 µl and 4 µl respectively, as shown in Table 3. During the reaction, Au^+^ ions were first reduced to Au due to it’s higher reduction potential than Ag. Then Ag^+^ gets reduced and gets deposited over the surface of Au and sort active sites for the growth process. Thus, with the increase of AgNO_3_, the catalytic effect of Ag^+^ gets sufficiently increased to form different shapes of bimetallic nanoparticles.

**Table 3:** The synthesis specifications of Au-Ag bimetallic nanoparticles with different proportions.

Sample ratio	HAuCl_4_ (10 mM)	AgNO_3_ (10 mM)	AA (100 mM)
Au-Ag (10:1)	20 µl	2 µl	4 µl
Au-Ag (10:2)	20 µl	4 µl	4 µl
Au-Ag (10:3)	20 µl	6 µl	4 µl
Au-Ag (10:4)	20 µl	8 µl	4 µl
Au-Ag (10:5)	20 µl	10 µl	4 µl
Au-Ag (10:6)	20 µl	12 µl	4 µl
Au-Ag (10:7)	20 µl	14 µl	4 µl
Au-Ag (10:8)	20 µl	16 µl	4 µl
Au-Ag (10:9)	20 µl	18 µl	4 µl
Au-Ag (10:10)	20 µl	20 µl	4 µl

### Fabrication of SERS substrate

To use the synthesized nanoparticles for fabricating SERS substrates, these were transferred on to cleaned glass slides by drop-casting of 10 µl nanoparticle solution. The glass slides were chemically cleaned using piranha solution with standard cleaning procedure, followed by plasma cleaning for 15 minutes using a plasma cleaner (Harrick Plasma, PDC-001). The glass plates containing the dropcasted nanoparticle solution was dried inside a vacuum desiccator at room temperature for 30 minutes.

### Characterization techniques

The morphology of the synthesized bimetallic nanoparticles was investigated using a field emission scanning electron microscope (Carl Zeiss, Gemini SEM 500), the crystal structural phase information of nanoparticles was examined using X-ray diffractometer (Rigaku, TTRAX-III), and the optical properties of nanoparticles were investigated using UV-Visible spectrophotometer (Perkin Elmer, Lambda 35).

### Binding of Raman probe molecules over SERS substrate

To evaluate the SERS activity of Au-Ag bimetallic nanoparticles, a parent solution of MB (dissolved in H_2_O) at 10^−3^ M was prepared and was diluted further to obtain different lower concentrations up to 10^−15^ M. For SERS activity, the analyte molecules were dispersed on fabricated SERS substrates by drop-casting of 10 µl MB solution of concentration ranging from 10^−6^ M to 10^−15^ M. Similarly, analyte solution of CV are also prepared through same method. The conventional SERS measurements were performed using He-Ne laser of wavelength 632.8 nm with an objective lens (NA: 0.45) attached to the upright Raman microscope (Seki Tech, STR-750). The Raman scattered light from the molecules was collected in the backscattering geometry using the combination of a 0.750 m imaging triple grating monochromator (Princeton Instruments, Acton SP2750i) and charge-coupled device camera (PIXIS-256E). All spectra were acquired with exposure time of 2 s and number of accumulations with 15 scans.

### Numerical methodology

To estimate the local electric field spectra of bimetallic nanoplasmonic structures, 3D modelling based on finite element method (FEM) were performed using COMSOL Multiphysics commercial software package installed in computing server with an Intel Xeon, 2.67 GHz 64-bit processor, 42 GB RAM. The frequency domain FEM solver in RF module was used for these study and surrounding medium has been chosen as air. Plane wave with unit electric field was used to excite localized surface plasmons. The electric field of incident light was chosen parallel to the dimer axis in all cases. The perfectly matched layer of suitable dimensions was used to eliminate undesired back reflections.

## Supplementary information

Supplementary information

## Data Availability

Authors confirm that all relevant data are included in the paper and its supplementary information file.
