# Supplementary material for: Enhancement of Single Molecule Raman Scattering using Sprouted Potato Shaped Bimetallic Nanoparticles
Source: Sci Rep. 2019 Jul 24;9:10771. doi: 10.1038/s41598-019-47179-4 (PMC6656737; doi:10.1038/s41598-019-47179-4)
Supplement: Supplementary file 1 — Supplementary information [file 41598_2019_47179_MOESM1_ESM.pdf]

Supplementary material: Enhancement of Single Molecule Raman Scattering using Sprouted Potato Shaped Bimetallic Nanoparticles

R. V. William, G. M. Das, V. R. Dantham* and R. Laha

Department of Physics, Indian Institute of Technology Patna, Bihta, India - 801103

*Corresponding author: dantham@iitp.ac.in

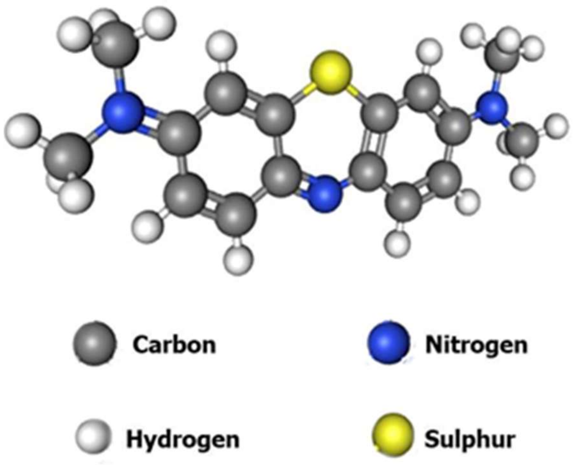

Figure S1. Molecular structure of MB.

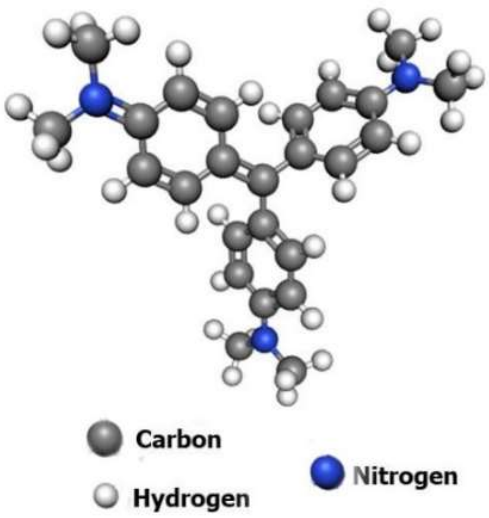

Figure S2. Molecular structure of CV.
